# Fit of cobalt–chromium implant frameworks before and after ceramic veneering in comparison with CNC‐milled titanium frameworks

**DOI:** 10.1002/cre2.9

**Published:** 2015-10-26

**Authors:** Per Svanborg, Victoria Stenport, Alf Eliasson

**Affiliations:** ^1^ Department of Prosthetic Dentistry/Dental Materials Science, Institute of Odontology, Sahlgrenska Academy University of Gothenburg Gothenburg Sweden; ^2^ Department of Prosthetic Dentistry Postgraduate Dental Education Center and Faculty of Medicine and Health, Örebro University Örebro Sweden

**Keywords:** Computer‐aided design, dental marginal adaptation, dental prosthesis, implant‐supported, metal ceramic alloys

## Abstract

Computer‐aided design/computer‐aided manufacturing fabrication of implant‐supported frameworks is a standard procedure, and the use of ceramic‐veneered cobalt–chromium alloys is increasing. However, no data are available concerning the precision of fit of these frameworks and the impact on the fit of the veneering procedure. The aim of this study was to evaluate the fit of computer numeric‐controlled‐milled cobalt–chromium and titanium implant frameworks for edentulous maxillas, provided with six implants. An additional aim was to evaluate the effect of ceramic veneering on the fit of the cobalt–chromium frameworks. Ten stone casts simulating an edentulous maxilla provided with six dental implants and abutments were produced. One computer numeric‐controlled‐milled cobalt–chromium framework and one titanium framework were fabricated for each stone cast. Each stone cast and corresponding titanium and cobalt–chromium framework was measured with a coordinate measuring machine in the three‐dimensional (*X* axis, *Y* axis, and *Z* axis) directions. Both milled titanium and cobalt–chromium frameworks presented a good fit in the vertical plane (*Z* axis), 5.3 µm for titanium frameworks and 4.6 µm for the cobalt–chromium frameworks. The titanium frameworks showed a statistically significant smaller mean degree of misfit in the horizontal plane, *X* (5.0 µm) and *Y* (2.8 µm) axes as compared with the cobalt–chromium frameworks presenting a mean deviation of 13.5 µm in *X* axis and 6.3 µm in *Y* axis (*P* < 0.001). *After ceramic veneering* of the cobalt–chromium frameworks, the horizontal distortion significantly decreased from 13.5 to 9.7 µm in *X* axis (*P* = 0.007) and from 6.3 to 4.4 µm in *Y* axis (*P* = 0.017). The fit of both titanium and cobalt–chromium frameworks was very good. There were small but significant differences in fit between the titanium and cobalt–chromium frameworks, but the difference is of no clinical significance. The ceramic veneering resulted in a minor but significant improvement of the fit for the cobalt–chromium frameworks.

## Introduction

Initially, frameworks for implant‐retained bridges were cast in sections and soldered, or in one‐piece castings. The process of investing and casting implant frameworks is complicated and technique sensitive, usually resulting in misfit (Carr and Stewart 1993; de Torres et al. 2007; Karl et al. 2004). To overcome this problem, frameworks have been sectioned and soldered or laser‐welded vertically and horizontally. Horizontally sectioned and laser‐welded frameworks in titanium (Ti) and cobalt–chromium (CoCr) using the Cresco^TM^ method have worked well in clinical situations (Hedkvist et al. 2004; Hellden et al. 2003). According to Riedy and colleagues, laser‐welded frameworks in Ti had better precision compared with one‐piece cast frameworks (Riedy et al. 1997).

Early attempts to industrialize the manufacturing of implant frameworks include prefabricated sections that were laser‐welded. However, laser‐welded frameworks had more fractures and complications compared with cast gold‐alloy (Au) frameworks after 15 years (Ortorp and Jemt 2009). In the late 1990s, milled Ti‐frameworks using computer numeric‐controlled (CNC) milling machines were introduced. According to several studies, CNC‐milled Ti frameworks have a better fit compared with cast frameworks from different alloys, frameworks produced using the Cresco^TM^ method, and CNC‐milled zirconia (Abduo et al. 2011; Al‐Fadda et al. 2007; Hjalmarsson et al. 2010; Katsoulis et al. 2014; Ortorp et al. 2003; Takahashi and Gunne 2003). There has been a rapid development of digital technologies in dentistry (van Noort 2012) and according to a consensus statement, computer‐aided design/computer‐aided manufacturing (CAD/CAM) technologies have been successfully implemented into implant dentistry (Wismeijer et al. 2014).

Several authors have attempted to define “passive” fit with the acceptance of a certain degree of vertical misfit, ranging from 40 to 150 µm (Jemt 1991; Klineberg and Murray 1985), although there is yet no consensus. Others have tried to define “passive” fit as “Fit which is less than perfect, but the application of any external forces to produce a perfect fit has a negligible effect on the performance of the prosthesis” (Patterson 1996), or as “To provide passive fit or a strain‐free superstructure, a framework should, theoretically, induce absolute zero strain on the supporting implant components and the surrounding bone in the absence of an applied external load” (Sahin and Cehreli 2001). Although perfect accuracy is only achievable in theory (Sahin and Cehreli 2001), what is clinically accepted is still disputed. It is known that framework strain is affected by the vertical misfit (Abduo and Lyons 2012; Abduo et al. 2012); thus, clinically well‐fitting prostheses may still have considerable external preload (Smedberg et al. 1996).

The importance of passive fit relating to biological and technical complications is still debated and so far no study has produced frameworks with a passive fit (Eliasson et al. 2010). Contradicting results have been reported concerning the impact of misfit on the surrounding bone. A study using finite element analysis (FEA) as well as one experimental animal study have shown that the surrounding bone was negatively affected by prostheses with misfit and dynamic load situations (Duyck et al. 2001; Kunavisarut et al. 2002). However, other animal studies have shown that prostheses with misfit did not lead to biologic failure but may instead promote bone remodeling (Duyck et al. 2005; Jemt et al. 2000). In a clinical study on prostheses with different levels of misfit, no differences in marginal bone loss were reported (Jemt and Book 1996).

When considering technical failures, framework misfit has been claimed to be related to screw loosening and screw fractures, which according to two systematic reviews are the second and third most common complications, only veneer fractures being more frequent (Pjetursson et al. 2004; Pjetursson et al. 2007). According to a FEA study by Sertgoz, rigid materials should be chosen as superstructure for implant‐retained fixed dental prostheses (FDPs), in order to reduce the risk of technical complications (Sertgoz 1997). One of the most rigid material combinations available in implant dentistry today is CoCr with ceramic veneer, which demonstrated clinical results (biological and technical complications) comparable with an Au alloy over up to 18 years, according to a study by Teigen and Jokstad (Teigen and Jokstad 2012). Few porcelain chippings were reported in studies on ceramic‐veneered CoCr tooth‐supported FDPs and single crowns after 5 years (Ortorp et al. 2012; Svanborg et al. 2013), indicating that the material combination may be suitable for situations with high occlusal loading.

The fit of metal–ceramic prostheses may be influenced by the veneering process (Fonseca et al. 2003), although full arch implant‐retained Ti frameworks evaluated using a coordinate measuring machine (CMM) before and after ceramic veneering and cast three‐unit CoCr and Ti frameworks evaluated using the one‐screw fit test before and after simulated ceramic firings were not significantly affected (Ortorp et al. 2003; Tiossi et al. 2008). However, the fit of CNC‐milled CoCr frameworks for implant‐supported full arch prostheses in the edentulous jaw before and after ceramic veneering has not been studied.

The aim of this study was to evaluate the fit of CNC‐milled cobalt–chromium and Ti implant frameworks for edentulous maxillas, provided with six implants. An additional aim was to evaluate the effect of ceramic veneering on the fit of the CoCr frameworks.

The hypothesis was that the fit of CNC‐milled cobalt–chromium frameworks is similar to the fit of Ti frameworks and that it is unaffected by ceramic veneering.

## Material and Methods

### Fabrication of models and acrylic resin pattern

A model of an edentulous maxilla in type 4 stone was fitted with six implant abutment replicas (Ankylos, BalanceC, Balance Base Abutment 5.5, Dentsply, Friadent GmbH, Mannheim, Germany) and duplicated 10 times using duplicating silicone (Zhermack Duplication Silicone, Elite Double 32 Extra Fast, Zhermack SpA, Badia Polesine, Italy), type IV stone (Shera Hard Rock, Shera Werkstoff Technologie GmbH & Co., Lenförde, Germany) and Ankylos Balance Base retention copings. One acrylic resin pattern simulating a patient case (designed for ceramic veneering) was made from a tooth‐setup and used for fabrication of frameworks on abutment level.

### Fabrication of frameworks

One CNC‐milled CoCr (Starloy Soft, Dentsply, DeguDent, Hanau‐Wolfgang, Germany) framework and one Ti (commercially pure [CP], grade 4) framework were fabricated for each stone cast (Atlantis ISUS, Dentsply, Hasselt, Belgium), material composition (Table 1), resulting in 10 CoCr frameworks and 10 Ti frameworks for the 10 stone casts. The horizontal tolerance of the milling procedure was 5 µm.

**Table 1:** Alloy composition in percent (%).

Alloy	Ti	Fe	O	H	C	N	Co	Cr	W	Si	Mn	Nb
Titanium	Bal	>.50	>.40	>.10	>.10	>.05						
Starloy soft		7.5					54.1	20.0	16.4	1.5	0.3	0.2

### Measuring of models and frameworks

The stone casts and frameworks were sent to an independent measuring laboratory in Sweden (Mylab AB, Gothenburg, Sweden). The mating surfaces of abutment replicas and frameworks were measured with a CMM (Zeiss Prismo Vast, Carl Zeiss Industrielle Messtechnik GmbH, Oberkochen, Germany). Each stone cast and corresponding Ti and CoCr framework was measured with a scanning head equipped with a contact stylus, which could be positioned at any *x*, *y*, and *z* location within the CMM working space. The position and angulation of the center point of all abutment replicas and the corresponding framework fit surfaces were calculated using the measurements of the mating surfaces. The measuring machine and procedures are similar to those described by Örtorp et al. (Ortorp et al. 2003). Five repeated measurements of one stone cast and one framework were conducted to establish the precision of the CMM measurements. The standard deviations of the *x*, *y*, and *z* coordinates for the six implant positions were within ±2 µm.

### Analysis of fit

**Figure 1:**
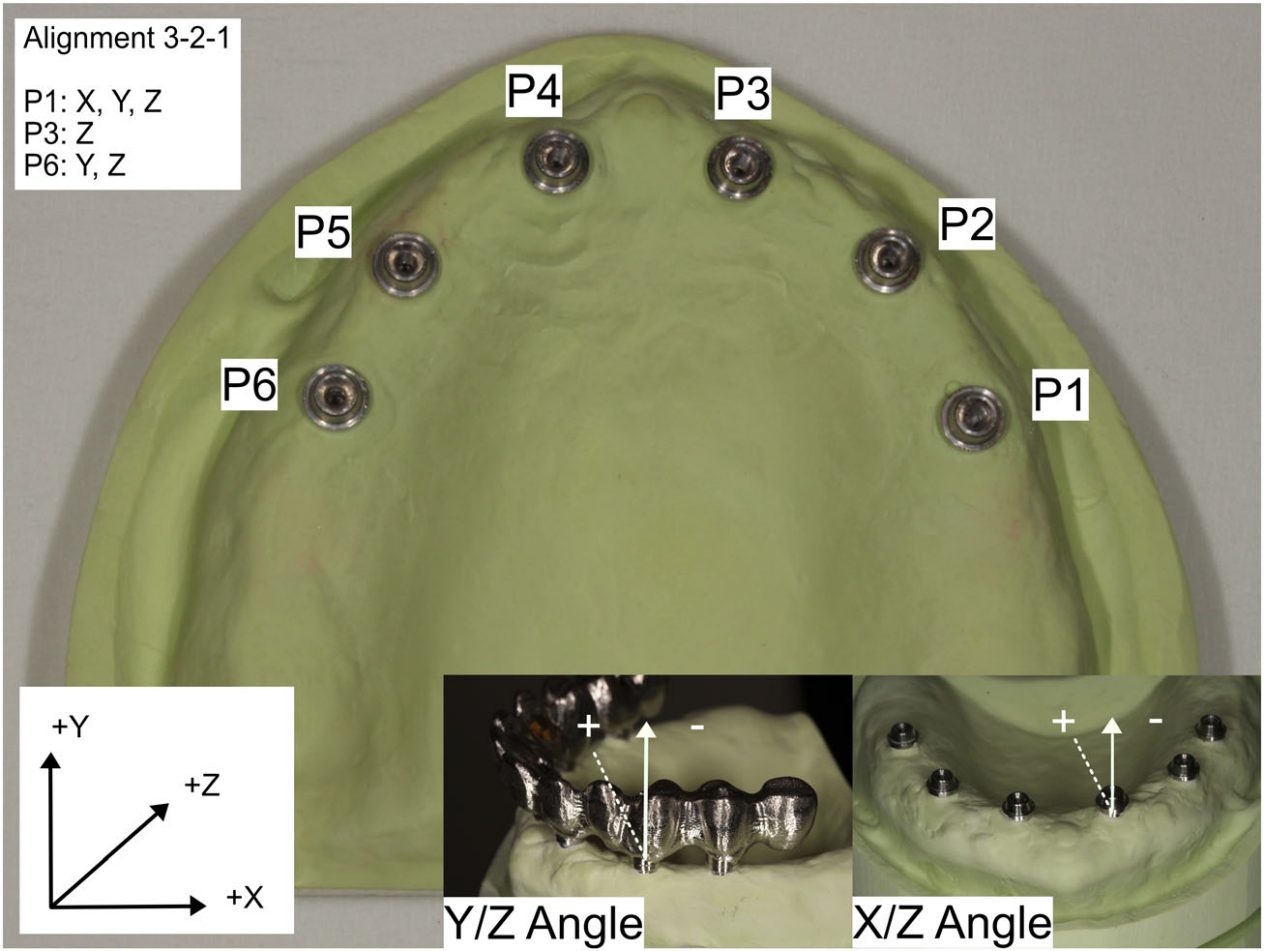
Master model with implant positions 1–6 and coordinate measuring machine measurement directions.

The distortion of frameworks was calculated and the data analysis regarding fit of the framework mating surfaces was calculated using the “least square method”, described by Bühler (Bühler 1981), where the position of the framework is superimposed in a “best fit” position onto the abutment replicas according to the center point positions. The three‐dimensional (*X* axis, *Y* axis, and *Z* axis) directions of displacement of the center points were calculated in micrometer in real and absolute figures. Furthermore, the three‐dimensional distance (3D) between the center points of the frameworks and the master model replicas was calculated for each individual cylinder using the formula (3D = √*x* ^2^ + *y* ^2^ + *z* ^2^). Additionally, the differences in angulation misfit were analyzed in *X*/*Z* angle and *Y*/*Z* angle for all implant positions. Also, the intersecting distances between the center points of implants in positions 1 and 6, positions 2 and 5, and positions 3 and 4 were measured and compared with the distances from the actual model, before and after ceramic veneering (Fig. 1).

### Ceramic veneering of cobalt–chromium frameworks

The CoCr frameworks were ground using carbide milling cutters and sandblasted with Al_2_O_3_ (110 µm/3–4 bar). The frameworks were thereafter sent to a commercial dental laboratory for ceramic veneering. The veneering procedure and firing cycles are described in Table 2 (GC Initial MC, GC Nordic AB, Älvsjö, Sweden). After veneering, the mating surfaces on the frameworks were blasted with 50 µm glass beads at 2–3 bar (Magma 50 µm, M‐Tec Dental AB, Malmö, Sweden) to remove the oxide. Thereafter, the frameworks were returned to the independent measuring laboratory for the same scanning procedure.

**Table 2:** Ceramic veneering of the CoCr frameworks. Firing cycles and materials. Furnace Ivoclar Programat P90.

*n*	Program	Material	End temp (°C)
1	Oxidation	N/A	950
1	Bonding	GC Initial Metalbond	980
2	Opaque	GC Initial MC Paste Opaque	960
2	Dentin	GC Initial MC Dentin GC Initial MC Enamel	905
1	Glaze	N/A	870

*n* = number of firing cycles.

### Statistical analysis

The spss (IBM spss v.22.0, Chicago, IL, USA) statistics software program was used for statistical analysis. The distribution was analyzed using box‐plots, and the non‐parametric Mann–Whitney *U* test was used to analyze the differences between the Ti and CoCr frameworks, and the Wilcoxon signed‐rank test for related samples was used to analyze the differences before and after ceramic veneering for the CoCr frameworks.

## Results

Both milled Ti and CoCr frameworks presented a good fit with a small mean deviation of 5.3 µm for Ti frameworks and 4.6 µm for the CoCr in the vertical plane (*Z* axis), the difference not being statistically significant (*P* = 0.481). However, the Ti frameworks had a statistically significant smaller mean degree of misfit in the horizontal plane, *X* (5.0 µm) and *Y* (2.8 µm) axes as compared with the CoCr frameworks presenting a mean deviation of 13.5 µm in *X* axis and 6.3 µm in *Y* axis (*P* < 0.001). The overall 3D distortion was slightly larger for the CoCr frameworks with a mean of 17.8 µm as compared with 9.0 µm for the Ti frameworks (*P* = 0.023) (Table 3).

**Table 3:** Mean distortion (SD) in micrometers of the center point of the frameworks presented with the master model as reference using least square method, in absolute figures.

Framework	*n*	/*x*/	SD	/*y*/	SD	/*z*/	SD	3D	SD
Ti frameworks	10	5.0	(1.5)	2.8	(0.6)	5.2	(2.4)	9.0	(1.5)
CoCr frameworks	10	13.5	(7.4)	6.3	(3.4)	4.6	(2.8)	17.8	(7.7)
CoCr frameworks veneered	10	9.7	(6.9)	4.4	(4.0)	4.9	(3.1)	13.7	(7.9)

*n* = number of frameworks.

The angular deviation was small for all frameworks with a mean deviation in *X*/*Z* direction and in *Y*/*Z* direction of less than 0.07° for CoCr frameworks and less than 0.06° for Ti frameworks, the difference not being statistically significant (Table 4).

**Table 4:** Mean deviation (SD) in angulation in decimal degrees of the mating surfaces of the frameworks using least square method, in absolute figures.

Framework	*n*	*X*/*Z* angle		*Y*/*Z* angle	
		Mean	SD	Mean	SD
Ti frameworks	10	.044	.030	.058	.020
CoCr frameworks	10	.061	.022	.067	.026
CoCr frameworks veneered	10	.074	.038	.068	.039

*n* = number of frameworks.

*After ceramic veneering* of the CoCr frameworks, the horizontal distortion decreased from 13.5 to 9.7 µm in *X* axis (*P* = 0.007) and from 6.3 to 4.4 µm in *Y* axis (*P* = 0.017). The difference in the vertical plane, 4.6 µm before and 4.9 µm after, was not statistically significant (*P* = 0.184). The decrease in the 3D distortion from 17.8 to 13.7 µm was statistically significant (*P* = 0.005) (Table 3). The angular deviation was slightly increased in the *X*/*Z* direction, although the difference was not statistically significant (Table 4).

The distances between the implant positions 1–6, 2–5, and 3–4 were measured before and after ceramic veneering and compared with the actual models. Before veneering, the framework arches were slightly smaller than the model with a mean difference in distance between implants 1 and 6 of 47 µm. This difference was reduced by the veneering procedure to 27 µm (*P* = 0.005) (Table 5). Statistically significant differences in distance before and after veneering were also recorded for positions 2–5 (*P* = 0.008) but not for positions 3–4 (*P* = 0.527).

**Table 5:** Mean deviation in distance in millimeters between the center points of the frameworks.

	Center point positions			
Framework	*n*	1–6	2–5	3–4
Model	10	40.548	30.898	11.781
CoCr frameworks	10	−0.047	−0.023	−0.002
CoCr frameworks veneered	10	−0.027	−0.017	−0.001

*n* = number of frameworks.

## Discussion

The aim of this study was to evaluate the fit of CNC‐milled CoCr and Ti implant frameworks for edentulous maxillas, provided with six implants, and to evaluate the fit of CoCr frameworks before and after ceramic veneering using a CMM equipment. Different techniques have been used to measure misfit of implant frameworks. With the CMM, not only the vertical misfit was evaluated but also the horizontal and angular misfit. However, one disadvantage was that the software might have superimposed the frameworks on the mating surfaces with a negative value resulting in a vertical misfit slightly less than the real one. Thus, the results should only be compared with other studies using the same measuring technique. The use of measuring microscopes for measuring vertical discrepancies will inevitably result in a larger registered misfit.

In the present study, the comparison of the two materials, Ti and CoCr, revealed small differences in fit. However, the distortion in the horizontal plane and 3D was statistically significantly smaller for the Ti frameworks, while the vertical distortion was comparable. The reason for this difference could be an effect of wear of milling tools, more pronounced in the CoCr group as a result of a tougher material to mill. The fit of the CoCr frameworks was affected by the veneering procedure in the horizontal plane (*X* and *Y* axes) but not in the vertical plane. The veneering procedure resulted in a slight decrease in the arch curvature, which reduced the distortion in the horizontal plane and in 3D. These differences were statistically significant.

The intersecting distance between the implant positions showed that the frameworks only were affected to a small extent by the veneering process. The distance between the terminal implants, positions 1 and 6, changed the most, which is reasonable. The hypothesis that the fit of CNC‐milled CoCr frameworks was similar to the fit of Ti frameworks while being unaffected by ceramic veneering was therefore rejected.

Usually, studies on implant frameworks are conducted on implants placed in the mandible, where the curvature of the frameworks is less pronounced. In the study by Örtorp et al., the distance between the terminal implants was 30.9 mm (Ortorp et al. 2003). In studies on frameworks for the maxilla, the arch is larger as in the present study, and the frameworks showed a distance between the distal implants (positions 1–6) of 40.5 mm. A similar distance was reported by Katsoulis et al. (Katsoulis et al. 2015), investigating frameworks fabricated for the maxilla with a distance of 40 mm between the terminal implants. Theoretically, the veneering process might therefore affect frameworks for the maxilla more than frameworks for the mandible. However, this has not been confirmed.

The effect of ceramic veneering of Ti (CP grade 2) frameworks on the fit of implant‐retained FDPs has been studied by Örtorp et al. (Ortorp et al. 2003), who used a CMM to analyze the fit, reporting no significant change in fit by the veneering process. These results were confirmed by Katsoulis et al. (CP grade 4) (Katsoulis et al. 2015), measuring the vertical fit using the one‐screw fit test and a scanning electron microscope. Tiossi et al. (Tiossi et al. 2008), comparing Ti (CP grade 1) and CoCr in three‐unit implant‐retained FDPs before and after simulated porcelain firings and using the one‐screw fit test and an optical microscope to measure vertical misfit, reported no significant differences. This is in accordance with the results for vertical misfit for the CoCr frameworks in the present study. Fonseca et al. (Fonseca et al. 2003) studied marginal fit of tooth‐retained single crowns after simulated porcelain firings, using a travelling microscope, and all three materials (CP Ti grade 2, Ti6Al4V alloy, and PdAg alloy) were negatively affected by the firing procedures, with significant increases in marginal discrepancies. The reason for this difference in recorded distortion after heat cycles could be the thin cervical margins on tooth‐supported crowns as compared with the thick framework dimension in implant FDPs.

The clinical relevance of misfit in implant‐retained FDP is debated, where some studies claim that misfit is detrimental to the surrounding bone, whereas others claim that it is favorable and stimulates bone remodeling (Duyck et al. 2001; Duyck et al. 2005; Jemt and Book 1996; Jemt et al. 2000; Kunavisarut et al. 2002). Concerning the technical relevance of misfit, several studies argue that FDP misfit is related to screw complications (Al‐Turki et al. 2002; Kallus and Bessing 1993; Worthington et al. 1987; Zarb and Schmitt 1990). Thus, it is clear that a good framework fit should be strived for.

With the introduction of CAD/CAM techniques in fabrication of implant frameworks, the fit of prostheses has improved. Although passive fit of implant frameworks has not been reached yet, the improvement especially in the vertical fit may result in a decrease of screw loosening events. FEA studies have shown that increased stiffness in frameworks with misfit increases the risk of mechanical failures (Abduo and Judge 2014). However, one can only speculate whether the difference in stiffness of material, CoCr as compared with Au and Ti, may have an impact on the risk of screw loosening for frameworks with minor misfit. According to an FEA study, the stress in the retaining screw decreased for stiffer framework materials with a vertical misfit of 10 and 50 µm (Bacchi et al. 2013). Earlier clinical studies have reported an increase of screw loosening in frameworks with a misfit larger than 150 µm. In the present study, frameworks had a mean vertical misfit of less than 10 µm. The present CAD/CAM technique used for framework fabrication produces frameworks with a fit superior to cast frameworks and may therefore contribute to further reduction in technical complications for implant‐supported FDPs.

From an esthetic point of view, metal ceramic prostheses are more tooth‐like than frameworks veneered with acrylics, especially after some years in the oral cavity. On the other hand, ceramic chipping is one of the major drawbacks with metal ceramic dental prostheses. Recent laboratory studies indicate that the bond between CoCr and the ceramic veneer is strong (Akova et al. 2008; Joias et al. 2008; Lombardo et al. 2010), and some clinical studies support this by reporting a low incidence of ceramic chipping on tooth‐supported FDPs fabricated from CoCr alloy (Ortorp et al. 2012; Svanborg et al. 2013; Teigen and Jokstad 2012).

The CMM that was used for fit measurements is considered highly accurate for the purpose of measuring fit for implant‐retained FDPs, with the possibility of measuring not only the vertical but also the angular and the horizontal distortion (Abduo et al. 2010; Jemt and Hjalmarsson 2012). However, all measurements are made in a laboratory, and the appliance cannot be used for measuring fit in the patient. In the future, a 3D analysis of fit of the framework in the clinic might be performed with an intraoral scanner provided with a quality control software. In a recent publication, Jokstad and Shokati used an intraoral scanner combined with a laboratory scanner and software to assess clinical misfit of implant‐retained FDPs (Jokstad and Shokati 2015).

During the ceramic veneering, the mating surfaces of the frameworks are exposed and subsequently oxidized when heated. The oxide layer that accumulates needs to be removed using glass bead blasting. This procedure may be a source of vertical misfit if not carried out correctly. In the present study, the difference in fit after ceramic veneering and subsequent glass bead blasting was minimal. The effect of the blasting procedure was therefore not considered a confounder.

The fit of both Ti and CoCr frameworks was good, and the ceramic veneering procedure resulted in an improvement of the fit for the CoCr frameworks as a result of less distortion mainly in the *X* axis. The slight decrease in arch curvature after veneering resulted in less horizontal misfit, which also reduced the 3D misfit from 17.8 to 13.7 µm. In the present study, the best‐fit method was used in the evaluation of fit. The same method was used on frameworks after an average of 19 years of clinical use, reporting an overall misfit of the frameworks of 150 µm (range 95–232 µm) and stating that the effect misfit had on the long‐term clinical outcome was minor (Jokstad and Shokati 2015).

## Conclusions

The fit of both Ti and CoCr frameworks was very good. There were small but significant differences in fit between the Ti and CoCr frameworks, but the difference is of no clinical significance. The ceramic veneering resulted in a minor but significant improvement of the fit of the CoCr frameworks.

## Conflict of interest

Mr. Svanborg has nothing to disclose.

Dr. Eliasson has nothing to disclose.

Dr. Stenport has nothing to disclose.
